# Supplementary material for: Multiple preferred escape trajectories are explained by a geometric model incorporating prey’s turn and predator attack endpoint
Source: eLife. 2023 Feb 15;12:e77699. doi: 10.7554/eLife.77699 (PMC10065801; doi:10.7554/eLife.77699)
Supplement: Figure 3—source data 1. [file elife-77699-fig3-data1.docx]

**Figure 3—source data 1.** Akaike information criterion (AIC) for 1–9 Gaussian mixture models to estimate the empirical ET distribution. The dataset and R code are available at Figshare (“Dataset1.csv” and “Source code 1.R”) (n=264 from 23 individuals)

| Number of peaks | AIC | ΔAIC |
| --- | --- | --- |
| **3** | 2768.8 | 0.0 |
| 4 | 2773.0 | 4.2 |
| 2 | 2777.8 | 9.0 |
| 5 | 2779.3 | 10.5 |
| 6 | 2780.5 | 11.6 |
| 7 | 2785.9 | 17.1 |
| 8 | 2791.2 | 22.3 |
| 9 | 2796.8 | 27.9 |
| 1 | 2996.6 | 227.8 |

The best model is shown in bold.
