# Supplementary material for: Multiple preferred escape trajectories are explained by a geometric model incorporating prey’s turn and predator attack endpoint
Source: eLife. 2023 Feb 15;12:e77699. doi: 10.7554/eLife.77699 (PMC10065801; doi:10.7554/eLife.77699)
Supplement: Table 2—source data 1. [file elife-77699-table2-data1.docx]

**Table 2—source data 1.** Widely applicable or Watanabe–Akaike information criterion (WAIC) for each model to estimate the relationship between the absolute value of the turn angle and the time required for a displacement of 10 or 20 mm from the initial position (n=264 and 263, respectively, from 23 individuals).

| Length of displacement | WAIC | ΔWAIC |
| --- | --- | --- |
| 10 mm |  |  |
| **Piecewise linear** | 1239.7 | 0 |
| Linear | 1259.0 | 19.3 |
| Constant | 1524.4 | 284.7 |
| 20 mm |  |  |
| **Piecewise linear** | 1543.3 | 0 |
| Linear | 1547.0 | 3.7 |
| Constant | 1689.7 | 146.4 |

The best models are shown in bold.
