# Supplementary material for: Multiple preferred escape trajectories are explained by a geometric model incorporating prey’s turn and predator attack endpoint
Source: eLife. 2023 Feb 15;12:e77699. doi: 10.7554/eLife.77699 (PMC10065801; doi:10.7554/eLife.77699)
Supplement: Table 3—source data 1. [file elife-77699-table3-data1.docx]

**Table 3—source data 1.** Comparison of the distribution of escape trajectories (ETs) between the prediction of the models in which *U*_pred_ was determined from the dummy predator speed per trial in the experiment [predator speed at the onset of escape response of prey, n=264 per simulation ×1000 times; mean predator speed to cover 75 % of the prey’s flight initiation distance (FID), n=257 per simulation ×1000 times] and experimental data (n=264 and 257) using the two-sample Kuiper test. Note that the sample size of the latter model is smaller than the total number of observations, 264, because the dummy predator did not move over 75 % of the FID in seven cases.

| Method to estimate the predator speed | Median Kuiper’s *V* | Median *P* | Rate of *P >* 0.05 |
| --- | --- | --- | --- |
| The speed at the onset of escape response of prey | 0.20 | < 0.01 | 0.03 |
| Mean speed to cover 75 % of the prey’s FID | 0.15 | 0.07 | 0.59 |
