# Supplementary material for: Multiple preferred escape trajectories are explained by a geometric model incorporating prey’s turn and predator attack endpoint
Source: eLife. 2023 Feb 15;12:e77699. doi: 10.7554/eLife.77699 (PMC10065801; doi:10.7554/eLife.77699)
Supplement: Table 3—source data 2. [file elife-77699-table3-data2.docx]

**Table 3—source data 2.** Comparison of the distribution of escape trajectories (ETs) between the model prediction (n=264 per simulation ×1000 times ×2 distances) and experimental data (n=264) using the two-sample Kuiper test.

| Distance for the fast-start phase | Median Kuiper’s *V* | Median *P* | Rate of *P >* 0.05 |
| --- | --- | --- | --- |
| 10 mm |  |  |  |
| With both *D*_attack_ and *T*_1_(\|*α\|*) | 0.10 | 0.63 | 0.99 |
| With *D*_attack_ and without *T*_1_(\|*α\|*) | 0.25 | < 0.01 | 0.00 |
| Without *D*_attack_ and with *T*_1_(\|*α\|*) | 0.17 | < 0.05 | 0.25 |
| Neither *D*_attack_ nor *T*_1_(\|*α\|*) | 0.28 | < 0.01 | 0.00 |
| 20 mm |  |  |  |
| With both *D*_attack_ and *T*_1_(\|*α\|*) | 0.11 | 0.44 | 0.96 |
| With *D*_attack_ and without *T*_1_(\|*α\|*) | 0.25 | < 0.01 | 0.00 |
| Without *D*_attack_ and with *T*_1_(\|*α\|*) | 0.15 | < 0.05 | 0.46 |
| Neither *D*_attack_ nor *T*_1_(\|*α\|*) | 0.28 | < 0.01 | 0.00 |

The distance for the fast-start phase was regarded as either 10 or 20 mm. *D*_attack_, distance between the prey’s initial position and the endpoint of the predator attack; *T*_1_(|*α|*), relationship between the absolute value of the turn angle and the time required for a 10- or 20-mm displacement from the initial position (i.e., the time required for the prey to turn).
